# Is intensive care the only answer for high risk pregnancies in developing nations?

**DOI:** 10.4103/0974-2700.70752

**Published:** 2010

**Authors:** Sukhwinder Kaur Bajwa, Sukhminder Jit Singh Bajwa, Jasbir Kaur, Kamaljit Singh, Jasleen Kaur

**Affiliations:** Department of Obstetrics and Gynaecology, Ram Nagar, Banur, India; 1 Department of Anaesthesiology and Intensive Care Ram Nagar, Banur, India; 2 Department of Biochemistry Gian Sagar Medical College and Hospital (GSMCH), Ram Nagar, Banur, India; 3 Department of Obstetrics and Gynaecology, Patiala, Punjab, India

**Keywords:** Intensive care, pregnancy induced hypertension, obstetrical hemorrhage, mechanical ventilation, multiorgan failure

## Abstract

**Background:**

Management of high risk obstetric patients.

**Aim:**

The present study was conducted to evaluate the primary causes of the admission of obstetric patients to Intensive Care Unit (ICU), the presence of co-morbid diseases, outcome of such patients, their survival rate as well as the factors which contribute to the maternal mortality.

**Settings and Design:**

A retrospective study was conducted in the Department of Obstetrics and Gynaecology and Anaesthesiology/ICU of our Institute.

**Materials and Methods:**

Sixty-one obstetric patients, who were admitted to ICU between 20 December 2006 and 31 January 2010, were evaluated for various factors responsible for their admission as well as their outcome.

**Statistical Analysis:**

At the end of study, the data were arranged systematically and subjected to statistical analysis using nonparametric tests and *P* value <0.05 was considered significant.

**Results:**

Majority of the 61 patients admitted in ICU were referred from the peripheral health centers, smaller nursing homes/hospitals and some even without proper primary care and mainly comprising uneducated and rural population. Hemorrhage, pregnancy induced hypertension, cardiac diseases, respiratory insufficiency and sepsis were the main causes for admission. A total of 18 patients among 61 died during their ICU stay in the hospital.

**Conclusions:**

In the developing countries, high risk pregnancy should be managed at peripheral centers with proper facilities, antenatal visits and timely referral. The intensive care help should be reserved for very high risk pregnancies with co-morbid diseases.

## INTRODUCTION

Maternal mortality is still on the higher side in developing countries like India in spite of so much of advancements in obstetrical critical care over the last few years.[1] Maternal mortality in developed countries like UK and USA is 7–8 per 100,000 live births as compared to more than 700 maternal deaths in most of the African countries. The figures for most of the South Asian countries are higher than 250 maternal deaths per 100,000 live births. Among these nations, India leads with an astounding statistics and accounts for more than 540 maternal deaths per 100,000 live births.[2] Though our national programs are continuously laying stress on the policies and urgency of decreasing maternal mortality and morbidity, especially in the rural population of our country, the established targets have not been achieved till now.

A major drawback in developing countries like India is the severe shortage of intensive care facilities as compared to the number of critically sick population. Obstetricians nowadays are facing new challenges, especially at tertiary care hospitals and at higher referral centers. This acquires all the more important dimensions in the background of extremely low ratio of Intensive Care Unit (ICU) specialists doctors/staff to manage such high risk patients. In most of the developing countries like India, majority of the population is distributed in the rural areas. The intellectual level of such rural patients and their relatives, their customs and traditions, lack of tertiary care facilities at villages, lack of transportation modes, as well as their financial status are the main contributory factors that account for the late admission of these patients to critical care units.[3,4] By the time these patients receive the intensive care services, it becomes quite late, and their condition gets deteriorated to a large extent. Some unfortunate patients are never able to receive the services of these higher centers, which adds to the already higher figures of maternal mortality and morbidity. These factors, combined with some structural and functional health problems, do not allow the developing countries to achieve targets in obstetrical critical care as those in developed countries.[5–7]

The higher admission rates of obstetric patients to critical care units are largely confined to urban population which is mainly due to their education level, awareness, realization of value of human life, changing social attitudes and sound financial condition. The increased awareness about the criticality of certain obstetric conditions among urban masses has further propelled the early admission of patients to tertiary care centers. With increasing load of critical obstetric patient, the role of obstetrician has become all the more challenging and dynamic and they have to keep themselves well acquainted with the ever changing needs of critically ill obstetric patients.[8] As a result, the role of obstetrician has extended beyond the boundaries of labor room and operation theatre to the world of intensive care. One big reason for this involvement in critical care settings is the fact that obstetricians are well versed with the physiology of fetus and mother as well as the effects of various treatment regimens and interventions on these.

The ICU of our institute is 12 bedded and well equipped with all modern gadgets and state of the art ventilators. The hospital is being run by a charitable trust and provides the most sophisticated tertiary care services to people of around 80–90 villages, at a very nominal cost. The hospital has synchronized its obstetric and other health services with national rural health mission and other government programs to provide free services to the rural obstetrical patient, which has come as a boon for these poor patients.

This study is aimed at a retrospective review of the obstetric patients who were admitted to ICU, the primary causes for their admission, the presence of co-morbid diseases, outcome of such patients, the types of various treatment regimens, their survival rate as well as the factors which contribute to the maternal mortality

## MATERIALS AND METHODS

After obtaining the approval of the Ethics committee of our institute, we undertook the retrospective study of obstetric patients who were admitted to ICU for one cause or another between 20 December 2006 and 31 January 2010. The following conditions were stressed in our study:

- primary indication of admission to ICU,
- gestational age of the patients,
- number of pregnancies,
- associated risk factors,
- system/organ involvement,
- types of treatment administered,
- total stay in ICU and
- outcome of the disease process.

The patient profile comprised 61 obstetric patients who were treated in ICU, irrespective of their gestational age, covering the postpartum period of 42 days as well. Mechanical ventilatory support became essential for 43 of these patients, while rest of the patients were treated without ventilatory support. Besides, the symptomatic and supportive treatment was carried out in close association with anesthesiologists and intensivists, on a patient to patient basis. During the stay in ICU, all the relevant investigations were carried out for diagnostic and therapeutic purposes, which ranged from routine to specialized tests. The ICU treatment comprised mechanical ventilation, monitoring of vital parameters, electrolyte and metabolic correction, parenteral and enteral nutrition, blood, platelets and plasma transfusion, hemodialysis and management of co-morbid diseases.

Strict and vigil monitoring was done for all patients during their stay in ICU, which included heart rate (HR), electrocardiogram (ECG), non-invasive and invasive blood pressure, end tidal carbon dioxide, pulse oximetry, central venous pressure monitoring, temperature, etc. At the end of the study, all the statistical data including patient demographics, indications for ICU admission, associated risk factors, organ involvement and patient outcome were organized systematically and subjected to statistical analysis with nonparametric tests like chi-square test. *P* value <0.05 was considered significant.

## RESULTS

During the specified period of the study, 6895 was the total number of deliveries that were recorded from the obstetrical ward, including the 61 obstetrical patients who were admitted in ICU. Most of the ICU admissions were unbooked cases which were referred from the peripheral health centers, smaller nursing homes/hospitals and some even without proper primary care.

The demographic profile of the patients is shown in Table 1.

**Table 1:** Demographic profile of the patients

Demographic characteristics	Number of patients	*P* value
Number of patients	61	–
Age of patients (mean ± SD)	26.78±5.64	
Median age in years	29	–
Parity status	Primigravida: 16 (26.23)	
	Multigravida: 45 (73.77)*	<0.05
Educational level	Illiterate: 28 (46)*	<0.05
	Up to 5^th^ class: 20 (33)*	<0.05
	Up to High School: 9 (15)	Ref-High School graduates
	>High school: 4 (6)	
Financial status (family income per month in Rs./$)	<2000/40–45 (74)**	<0.001
	2000/40 to 5000/100-14 (23)	Ref-income
	>5000/100-2 (3)	>Rs. 5000/$100 month
Antenatal booking	Booked: 6 (10)	
	Unbooked: 55 (90)**	<0.001
Location	Rural: 51 (84)**	<0.001
	Urban: 10 (16)	

* *P* < 0.05;

** *P* < 0.001; FIGURES IN PARENTHESIS ARE IN PERCENTAGE

The average mean age of these patients was 26.78±5.64 with a median value of 29 and a range of 19–38 years of age. There was a statistically significant correlation with regard to the gravida status (*P* < 0.05) as majority of patients were multigravida (73.77%). The referral of critically sick obstetric patients from other smaller hospitals was again a clinically higher significant entity (*P* < 0.001) as 90% of the cases were not registered. Educational status of these patients was another determinant which on statistical analysis revealed significant values (*P* < 0.05) as majority of these patients were either illiterate (46%) or had a very meagre education (33%). The financial well being of these patients followed the similar patterns as that of education as majority of our patients (74%) were below poverty line with an income of less than Rs. 2000 per month (*P* < 0.001). Also, 84% of the patients who got admitted in critically sick condition hailed from rural area, while only 16% of the population comprised urban society, which again turned out to be a highly significant value (*P* < 0.001) on statistical analysis.

The most common reasons for admission to ICU are shown in Table 2.

**Table 2:** Primary indication for shifting the patients to ICU

Primary indication	Number of patients [*n* (%)]
Hemorrhagic shock	28 (46)
Respiratory distress/failure	18 (29)
Heart disease/CVS instability	8 (13)
Neurologic disorders	7 (12)

CVS, CARDIOVASCULAR SYSTEM

The most common primary indication for admission to ICU was hemorrhagic shock (46%) followed by respiratory insufficiency (29%). Heart disease and cardiovascular instability was responsible for 13% of total obstetrical ICU admission, while neurologic disorders accounted for rest (12%) of the admissions

The most common underlying disorder for admission to ICU was haemorrhage, both antepartum and postpartum combined (40%), followed by pregnancy induced hypertension and eclampsia (23%) [Table 3]. Sepsis accounted for 18%, while prolonged or obstructed labor accounted for 10% of patients who got admitted in ICU. Coagulation disorders, renal failure and HELLP syndrome accounted for the rest of patients who got critically sick.

**Table 3:** Underlying disease/primary diagnosis of the patients

Underlying disease/primary diagnosis	Number of patients [*n* (%)]
Antepartum haemorrhage	17 (27.87)
Pre-eclampsia/eclampsia	14 (22.95)
Sepsis	11 (18.03)
Postpartum hemorrhage	7 (11.48)
Prolonged/obstructed labor	6 (9.8)
Renal failure	3 (4.9)
Coagulation disorder	2 (3.28)
HELLP syndrome (hemolysis, elevated liver enzymes and low platelets)	1 (1.64)

HELLP SYNDROME (HEMOLYSIS, LOW PLATELETS AND ELEVATED LIVER ENZYMES)

From Table 4 it is quite evident that there is long mean delay of 11.64 hours from the onset of complications to their referral to the higher centers. Most of the admissions to ICU were postpartum (80.33%) patients who already had complications, while 19.67% antepartum patients were either referred late or came with a history of severe hemorrhage, prolonged/ obstructed labor and its related complications. Two out of 12 antepartum patients died during the treatment at hospital, while the percentage of postpartum deaths were 32.65% [Table 5].

**Table 4:** Characteristics of patients’ admission to ICU

Characteristics of admission	Numerical values
Time interval between the onset of acute illness and admission to ICU in hours (mean ± SD)	11.64±3.56
Antepartum admissions	12 (19.67)
Postpartum admissions	49 (80.33)
Presence of co-morbid diseases	27 (44.26)
Death in antepartum cases	2 (16.67)
Death in postpartum cases	16 (32.65)

FIGURES IN PARENTHESIS ARE IN PERCENTAGE

**Table 5:** Treatment pattern of the patients in ICU

Treatment pattern in ICU	Number of patients [*n* (%)]	*P* value
Mechanical ventilation	Required: 43 (70.5)*	<0.05
	Not required: 18 (29.5)	
Ionotropic support	Required: 32 (52.45)*	<0.05
	Not required: 29 (47.55)	
Stay (days) in ICU (median)	6	
Outcome (death/survival)	18/43	

* *P* < 0.05; FIGURES IN PARENTHESIS ARE IN PERCENTAGE

Airway protection became necessary in 70% of the patients who were intubated and subsequently given mechanical ventilation, which on statistical analysis turned out to be a significant value (*P* < 0.05). A large number (53%) of patients did require the ionotropic support for the maintenance of hemodynamics. The total period of stay of all these obstetric patients in ICU ranged from minimum 1 day to maximum 21 days, with a median value of 6 days. We could not save 18 patients out of a total 61, many of whom had developed multiorgan failure [Table 6].

**Table 6:** Degree of organ involvement and its effect on mortality rate

Number of organs involved	Number of patients	Number of deaths and percentage of mortality [*n* (%)]	*P* value (ref -single organ involvement)
One	29	2 (7)	—
Two	13	3 (23)	—
Three	9	4 (44)	—
Four	4	3 (75)*	<0.05
Five+	6	6 (100)*	<0.05

* *P* < 0.05; FIGURES IN PARENTHESIS ARE IN PERCENTAGE; REF - COMPARISON OF THE MORTALITY RATES WITH REFERENCE TO SINGLE ORGAN INVOLVEMENT

Only two patients succumbed to their disease that had just a single organ involvement while binary organ dysfunction led to the deaths of 23% of the patients. Mortality increased significantly (*P* < 0.05) with progressive involvement of multiple organs as shown in the above table. Mortality rate increased to 44% with three, 75% with four and 100% deaths with five or more organs getting involved in the disease process. The most commonly involved organ system was CVS, followed by pulmonary tree, hematological system, nervous system, renal, hepatic and other organs. The hepato-renal involvement proved to be highly fatal.

## DISCUSSION

The ratio of maternal mortality to morbidity is a very good health predictor of health care delivery system. The study has thrown light on one very basic factor, that is, if high risk obstetric patients are managed at tertiary hospitals with all the advanced clinical facilities and equipment, maternal mortality and morbidity can be reduced to a large extent. Our present study has found quite a similar pattern with other such studies indicating the criteria for admission to ICU.[6,7,9–11] In our study, the education level of not just the patients but also the relatives determines the time, type and level of health care sought by them. To add insult to the injury, there were other contributing factors to their deterioration like poor financial status, transportation facilities, poor rural health infrastructure and customs and traditions of local community. One important factor which has been quite prevalent in our society is the preference of male baby, which has led to a large number of abortions at an advanced stage of gestation, thereby increasing the female feticide and subjecting the “poor” mother to many risks to her life by misuse of abortion services. One of the patients had delivered a female baby outside the hospital and had also consumed poison due to such related factors as she was shocked to see a female baby for the fourth consecutive time. The present day scenario in developing countries like India depicts a trend toward a still higher maternal mortality and morbidity due to all the previously discussed factors in rural population and increasing maternal age in career-oriented women, stressful lifestyle, lack of exercise and obesity, and preference for operative interventions in the urban obstetric patients. These factors responsible for such intensive care admissions are quite different from those in the developed world.[12–14]

The patients exhibiting the clinical picture of pre-eclampsia/ eclampsia, pulmonary edema, seizures, aspiration, congestive cardiac failure, etc. invariably did require ventilatory support in addition to the supportive treatment for the clinically presenting symptoms. The intervention strategies designed for the treatment of our patients were quite comparable with the methodologies employed by other authors.[7]

The infrastructure for intensive care treatment in our country is still at infancy stage due to acute shortage of ventilators and specialist doctors/staff. Ignorance and poor financial status are some of the other responsible factors. The absence of uniform specialist medical service and lack of proper medical facilities have immensely contributed to increasing the risk to our obstetric patients, which otherwise would have been properly managed at tertiary care health centers with timely interventions from the beginning itself. The government may have launched so many programs aiming at safe motherhood and child survival, but they are poorly implemented at the grass root level, the reasons for which are many but are out of scope for discussion in this article.

Had the patients undergone ultrasonography examination at appropriate health centers, few disorders like placenta accreta/ percreta, ectopic rupture and heterotopic pregnancy could have been timely diagnosed. The major reasons for ICU admission like hemorrhage, hypertensive disorders and heart diseases would have been prevented had the proper treatment been started during mid pregnancy. The hemorrhagic disorders most commonly occurred as a result of uterine atony due to prolonged or obstructed labor, retained products as well as due to iatrogenic causes, reflecting our poor obstetrical rural infrastructure and facilities and all these results are quite consistent with those of other such studies.[7,8,15,16] Provision of safe blood supply is an integral part of our national programs directed at improving safe motherhood practices. Though there was no shortage of blood and blood products in our blood bank, the patients who died of hemorrhage were the ones who came very late to our institution. In spite of all the resuscitative facilities available, they died due to their irreversible clinical disease entity as evidenced by the dehydration, respiratory distress, severe metabolic acidosis, electrolyte imbalance, sepsis and shock. The hemorrhagic shock contributed toward development of respiratory distress as well as failure. The other major contributors to respiratory failure were sepsis, pulmonary edema, renal failure and cardiogenic causes. The multiorgan involvement and failure does increase the maternal mortality[1,11,17,18] which has been very significant in our fact-finding where 5 or more than 5 organ involvement led to 100% mortality. Maternal mortality and morbidity due to infections (15%) is quite prevalent in developing countries and our results are very much consistent with these facts.[19] The common underlying causes of sepsis in patients were puerperal sepsis, sepsis due to perforation of uterus and intestines, and improper sterilization methods during delivery at periphery.

This study has again found the already present limitations in our health infrastructure pointing toward the improper obstetric care, untimely referral system, customs and traditions, lack of awareness, negligence in implementation of national programs and so on. The timely referral of such patients to tertiary care centers with ICU facilities does improve the outcome of obstetric patients.[20,21]

There is no absolute scoring pattern which can be applied to assess the severity of critically ill obstetric patients because some methods underestimate while others overestimate the severity of such patients and it is mainly because of grossly altered physiological state due to pregnancy.[18,22,23] Though we commonly employ Acute physiological and Chronic Health Evaluation II (APACHE II) score to assess the severity and prognosis, other scores like Glasgow Coma Scale and different scores related to individual organ dysfunctions are far better predictors of prognosis and severity when measured in combination.

The results have thrown light on some very significant aspects in our study, which are the educational level and poor economic status of the patients, as large number of ICU admissions comprised either uneducated or poorly educated patients as well as patients belonging to poor economic strata. The socioeconomic status, educational level and antenatal visits can greatly predict the effects on obstetric complications and outcome.[24] Most of the patients in our study were unregistered, poor, uneducated and never received antenatal care and advice, which contributed to a large extent in increasing the maternal mortality and morbidity. These findings are in quite contrast to the characteristics of ICU admission of obstetric patients in advanced and developed nations. This is mainly because of the sociocultural and economic differences in developed and developing nations, which govern the characteristics and different patterns of critical obstetric care in these countries.[3,4,6–8]

## CONCLUSIONS

The pregnant patients with co-morbid diseases like hypertension and cardiovascular diseases, respiratory diseases, hepato-renal involvement, any systemic disease should be carefully monitored and regularly followed up and must be properly educated during the antenatal visits about the potential complications. Improvement is required at the peripheral health centers for proper antenatal care, early treatment/advice for related medical conditions and timely referral of high risk pregnancies to the tertiary care centers for advanced treatment. The national programs concerned with safety of mother and child have to be re-strengthened at the grass root level. Involvement as well as awareness of masses, in the background of local customs and traditions, is an essential pre-requisite to decrease the figures of maternal mortality and morbidity. More fellowship programs should be started to involve the doctors of various specialties into the mainstream by imparting them the knowledge and the essentials of intensive care, which will at least tide over the acute shortage of specialist intensive doctors and staff. Lucrative incentives should be provided to the specialists who are posted in rural centers. Regular visits by the health officials are required for the adequate upgradation and maintenance of health facilities in these centers. The work should be visible not only in the census but in reality as well. Adequate training centers should be opened for education of paramedical and other health workers. Advanced diagnostic facilities should be provided in such a manner at a particular center that it covers the maximum population of not only that region but also the surrounding areas as well.

After considering all the causes and consequences of this major issue, we conclude that high risk pregnancy should be managed at peripheral centers with proper facilities and timely referral. The intensive care resources should be reserved for very high risk pregnancies with co-morbid diseases especially related to cardiovascular, respiratory and hepato-renal diseases. The role of government agencies acquires significant dimensions and efforts should be made by the government to involve the corporate sectors, non government organizations and other agencies in delivering better obstetric health care, especially at peripheral health centers.
